# Comparison of alternative mixture model methods to analyze bacterial CGH experiments with multi-genome arrays

**DOI:** 10.1186/1756-0500-7-148

**Published:** 2014-03-14

**Authors:** Liliana Sofia Cardoso, Cláudia Elvas Suissas, Mário Ramirez, Marília Antunes, Francisco Rodrigues Pinto

**Affiliations:** 1 Centro de Química e Bioquímica, Faculdade de Ciências, Universidade de Lisboa, Lisboa, Portugal; 2 Centro de Estatística e Aplicações, DEIO, Faculdade de Ciências, Universidade de Lisboa, Lisboa, Portugal; 3 Instituto de Microbiologia, Instituto de Medicina Molecular, Faculdade de Medicina, Universidade de Lisboa, Lisboa, Portugal

**Keywords:** Microarray, Comparative genomic hybridization, Data analysis

## Abstract

**Background:**

Microarray-based comparative genomic hybridization (aCGH) is used for rapid comparison of genomes of different bacterial strains. The purpose is to evaluate the distribution of genes from sequenced bacterial strains (control) among unsequenced strains (test). We previously compared the use of single strain versus multiple strain control with arrays covering multiple genomes. The conclusion was that a multiple strain control promoted a better separation of signals between present and absent genes.

**Findings:**

We now extend our previous study by applying the Expectation-Maximization (EM) algorithm to fit a mixture model to the signal distribution in order to classify each gene as present or absent and by comparing different methods for analyzing aCGH data, using combinations of different control strain choices, two different statistical mixture models, with or without normalization, with or without logarithm transformation and with test-over-control or inverse signal ratio calculation. We also assessed the impact of replication on classification accuracy. Higher values of accuracy have been achieved using the ratio of control-over-test intensities, without logarithmic transformation and with a strain mix control. Normalization and the type of mixture model fitted by the EM algorithm did not have a significant impact on classification accuracy. Similarly, using the average of replicate arrays to perform the classification does not significantly improve the results.

**Conclusions:**

Our work provides a guiding benchmark comparison of alternative methods to analyze aCGH results that can impact on the analysis of currently ongoing comparative genomic projects or in the re-analysis of published studies.

## Findings

### Background and purpose

This manuscript is an update of a previous work [1] on the analysis of microarray based comparative genomic hybridization (aCGH) using multistrain arrays. aCGH is a tool used in the investigation of the genetic content of closely related microorganisms and screening for virulence factors [2,3].

The idea behind this technology is to generate microarrays from sequenced genomes, and then hybridize genomic DNA from other sources to these arrays. The aim is to detect similarities and differences in genomic content through the characterization of test and control samples given the genes common to both samples and those that are specific to the control sample [2].

In two color aCGH experiments, the control sample is composed of DNA from one or more strains with sequenced genomes represented in the array. The test strain is composed of DNA from a non-sequenced strain. Each sample is labeled with a different fluorochrome and hybridized to the microarray [1].

The analysis of aCGH experiments aims to classify the genes as present or absent. Many approaches have been applied [2,4-7], but most of these methods base their results on the logarithm of the intensity ratio (log-ratio or LR = log_2_ (T/C)) of both fluorescent signals (test (T) and control (C)). Genes present in both genomes will give a clear fluorescence signal in both channels (LR close to 0), and absent genes in the test sample are expected only to have a signal in the control channel (negative LR).

Concerning the control used when arrays are designed with multiple sequenced genomes, there is no consensus about the best option. Some studies have used a single strain control [8-11] while others adopted a mixed control approach [6,12]. Some of the studies argue that a multi-strain mix control, while generating a control signal for every spot on the microarray, leads to an increased signal intensity of core genome spots (that represent genes present in all strains) in the control channel and can complicate data analysis based on ratios [9,11]. In order to clarify these approaches, we previously performed a comparative study with a multi-strain array for *Streptococcus pneumoniae*[1], covering the genomes of three sequenced strains: TIGR4 (T4), R6 (R6) and G54 (G54). We used two different controls: a single strain (T4) and an equimolar mix of the three strains represented in the array (T4 + R6 + G54) and hybridized them with the tests strains (R6 and G54). We concluded that the use of a single strain control increases the error rate in genes that are part of the accessory genome, where more variation across unsequenced strains is expected, justifying the use of the mix control. This conclusion was derived from the comparison of the discriminatory power of the LR values, using the known presence/absence classification for each gene. No statistical method was used to estimate the best threshold LR value to divide present from absent genes. This step is necessary in the real application scenario, were the genome sequence of the test strain is not available. The present work complements our previous study by applying an expectation-maximization (EM) algorithm to estimate the best \threshold for the same experimental results. We will re-validate our previous conclusions about the choice of control and optimize several processing steps of microarray data analysis. In particular, we will evaluate the impact of the logarithm transformation and the normalization steps, which are common steps in analysis of expression microarrays and in CGH analysis. We also wish to test if using an inverse ratio (C/T instead of T/C or log_2_ (T/C)) provides better results with the EM algorithm. In the traditional ratio the present signals distribute around 1 and the absent signals are compressed between 0 and 1. The logarithm transformation alleviates this compression, but we hypothesize that the two classes of signals can be further separated if the C/T ratio is used.

### Experimental data

Part of the data analyzed in this study was obtained with a multi-strain *Streptococcus pneumoniae* CGH microarrays already analyzed in a previous study [1]. The array was designed at PFGRC/JCVI (*Streptococcus pneumoniae* Version 5), with 3425 oligonucleotide probes covering the genomes of three pneumococcal strains: R6, T4 and G54. Each probe was 70 base long and replicated four times in each array. The annotation file provided by the array manufacturer was used to define which gene, from any of the three reference genomes, was represented by which probe. For each different pair of test strain and control, four replicates were done, resulting in a total of 16 hybridizations. The four different hybridizations were R6 versus T4, R6 versus MIX (T4 + R6 + G54), G54 versus T4 and G54 versus MIX (T4 + R6 + G54). Dye swaps were applied in each set of four replicates. Microarray hybridization raw data was deposited in the ArrayExpress public database with accession number E-MEXP-1390.

The images of the microarrays were analyzed using Feature Extraction 9.1 software (Agilent Technologies, Palo Alto, CA). For each spot the signal was background corrected by subtracting the minimum feature signal in the array. The intensity-specific bias was removed through loess global normalization. The resulting spot average pixel intensities were used to compute several signal ratio measures: the test/control signal ratio (T/C) and the corresponding logarithm signal ratio (log (T/C)), and the control/test signal ratio (C/T), before and after normalization. Since each gene was spotted four times per array, data retrieved from each of the valid spots were averaged for a particular gene.

Additional data analyzed in this study was obtained from a published dataset [2] using a *Staphylococcus aureus* array (PFGRC/JCVI *Staphylococcus aureus* Version 5). Raw data files were imported from ArrayExpress (E-MEXP-2007). The hybridizations with a test strain represented in the array design were selected. This selection allowed the definition of which gene was present or absent in the test sample by using the information available in the array annotation files provided by the manufacturer. All the hybridizations used a single strain control. The analysis was conducted using only the probes representing a gene present in the control sample. Raw data files were generated by GenePix image analysis software. For each probe, the F633 median and F532 median were used to define the T and C signals.

Instead of hybridizations where the test strain is sequenced, we could have tested the different analysis methods with microarray results of genes that were confirmed by PCR assays. Although these assays are common in published studies, they are not well suited for our purpose. In each dataset the number of genes confirmed with PCR is normally low. We would have to analyze a high number of experiments to achieve a reasonable statistical confidence on the results. Additionally, PCR results may disagree with microarray result, even if both are physically correct. If the array is based on short oligos, that sequence can be conserved while the regions targeted by the PCR primers can be divergent (or the other way around). Moreover, the selection of genes for PCR verification is not random. Genes can be selected because they are biologically more interesting, or because they are more likely to confirm the microarray results. This bias would make it more difficult to see any accuracy differences between C/T and T/C ratios using genes verified by PCR.

### Multi strain spot signal correction

The *Streptococcus pneumoniae* microarrays were designed with three reference strains. The control sample can be a mixture of DNA of the same three strains. Thus, the microarrays where the samples are hybridized contain sequences that identify genes in one strain (group 1), two strains (group 2) or three strains (group 3). According to our previous study [1] when the mix control is used and the gene is present in the test strain, it is expected that the ratio of intensities T/C should be 3 if the gene is present in one strain (group 1), 3/2 if the gene is present in two strains (group 2) and 1 if the gene is present in three strains (group 3). When the gene is absent of the test sample, it is expected that T/C reach values close to 0. So we chose to analyze corrected T/C values by multiplying the factors, 1, 2/3 or 3, according to each gene group (1, 2 or 3, respectively). To obtain C/T corrected values the inverse factors are applied. When the single strain (T4) control is used, there are also two groups of spots: some contain sequences that identify T4 genes (group 1) while others contain sequences that do not identify T4 genes (group 2). In this case we only need to correct the signal for the group 2. Here, to calculate the signal ratio, the value of C is exchanged by a surrogate value that is the average of the C values for group 1 spots. All these corrections allow the application of the same classification algorithm to all spots in the array.

### Present/absent classification

For each different signal ratio measure we applied the Estimation-Maximization (EM) algorithm to fit mixture models and to classify genes as present or absent. A mixture model is a convex combination of probability distributions that allows modeling data sets composed by different subsets, each of which is modeled by its own distribution. The mixture models used in this study were: the Normal-Uniform (NU) model and Gamma-Gamma (G) model. The first considers that signal ratios from present genes are shaped by the Normal distribution, while the missing genes are modeled by the Uniform distribution [13]. The Gamma-Gamma mixture model assumes that the signal ratio follows a Gamma distribution both for present and for absent genes, although with different parameters [14].

The EM algorithm is an efficient tool in the estimation of mixture model parameters and classification of each gene in one of two distinct groups. Iteratively, it finds the parameters of both distributions in the mixture model that maximize the likelihood of obtaining the given observations. At each step it computes for each gene the probability that it belongs to the present gene distribution. After the algorithm converges to a stable parameter set, each gene is considered present if the probability that it belongs to the present gene distribution is greater than 0.5.

In this work the EM algorithm successfully converged in all occasions. The G model was not fitted to log(T/C) ratios because they present negative values, for which the Gamma distribution is not defined. Log(C/T) ratios were not evaluated because they have the symmetrical value of the log(T/C) ratios. As such, similar but symmetric fits would be expected for both ratios, yielding the same classification accuracies.

### Evaluation of classifications

The produced classifications were compared with the known classification. For each classification we identified each gene as a true positive (TP) or true negative (TN) if the EM classification agreed with the known genome, and as a false positive (FP) or false negative (FN) if the EM classification did not agree. A gene is considered positive (either TP or FP) when the EM classification predicted that gene to be present. With the number of TP, TN, FP and FN we can calculate the accuracy (Acc), that is, the proportion of correct results.

$$\mathrm{Acc}=\frac{\mathrm{TP}+\mathrm{TN}}{\mathrm{TP}+\mathrm{TN}+\mathrm{FP}+\mathrm{FN}}$$

To compare the results obtained with the mix and the single strain control, Wilcoxon rank sum tests for independent samples were used to detect significant differences in the respective average accuracies, obtained from 8 independent arrays for each control choice. To compare the results obtained with different signal ratios of mixture models, Wilcoxon signed rank tests for paired samples were used, since the variant methods were applied to the same replicate arrays. Differences were considered significant when p <0.05.

### Results

**Figure 1:**
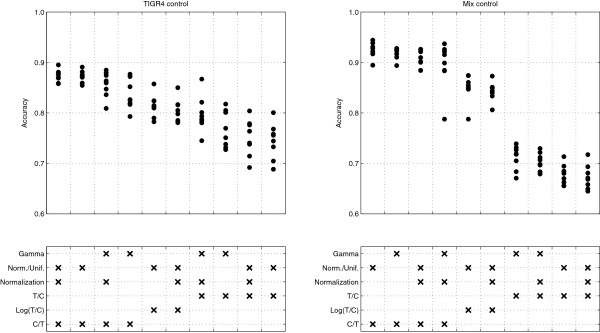
**Accuracy for the methods of analysis applied.** Each dot represents one *Streptococcus pneumoniae* array. The left plot represents the 8 arrays using the TIGR4 control, and the rights plot the 8 arrays using the mix control. The bottom boxes along the x-axis identify the different combinations of methods tested: the mixture model is either based on the Gamma distribution (Gamma) or in the Normal and Uniform distributions (Norm./Unif.); ratios can be normalized with the loess procedure (Normalization); and the signal ratio can be Test over Control signal (T/C), logarithm of the Test over Control (Log(T/C) or Control over Test (C/T). The order of the different method combinations is defined by the resulting mean accuracy. Each method combination was applied to all the 16 arrays.

Figure 1 shows the accuracy values for the pneumococcal arrays. The combinations of methods that have been tested were ordered according to the mean accuracies achieved. The highest accuracies were obtained with the mixed strain control, using the C/T ratio without normalization or logarithm transformation and fitting a normal-uniform mixture model.

To compare the performance of both control types, the best analysis options for each kind of control were used. The mixed strain control (with C/T ratio, NU model and without normalization) reaches accuracies that are on average 0.05 superior (p < 0.001) to the single strain control (with normalized C/T ratio and NU model). This accuracy difference may translate in more 50 to 350 correctly classified genes, considering bacterial genomes with 1000 to 7000 genes.

The impact of normalization and the mixture model choice are not clear from the analysis of Figure 1. Results obtained with respect to the mixture model used are contradictory when the ratio is C/T or T/C. NU mixture model performs better with C/T ratios, while G mixture model produces higher accuracies when applied to T/C ratios.

**Figure 2:**
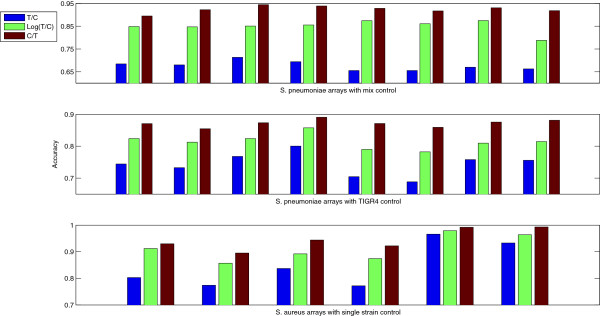
**Array-wise accuracy values with different signal ratios.** The blue bars correspond to Test over Control ratio (T/C), green bars to the logarithm of the Test over Control ratio (Log(T/C) and the brown bars to the Control over Test ratio (C/T). Each cluster of three bars was obtained from the same array. The top row shows results for *Streptococcus pneumoniae* arrays with the mix control, the center row for *S. pneumoniae* arrays with the TIGR4 control and the bottom row for *Staphylococcus aureus* arrays using a single strain control. In all arrays a Normal-Uniform mixture model was adjusted to non-normalized signal ratios.

The choice of the ratio has a clear impact in the resulting accuracies. Independently of the control type, C/T ratios perform better than T/C ratios, and log(T/C) ratios present intermediate accuracies (Figure 1). To confirm this pattern, we re-analyzed a set of 6 CGH hybridizations where genomic DNA from sequenced *Staphylococcus aureus* strains was hibridyzed with a *S. aureus* microarray using a single strain control. Although these arrays are also based on 70-mer probes, they were designed for a different species and analyzed with different image analysis software. Figure 2 shows for each of the 16 pneumococcal, plus the 6 *S. aureus* microarrays the accuracies obtained by using the T/C, Log(T/C) or C/T ratio. The pattern observed in Figure 2 is extremely consistent across arrays. The C/T ratios lead to 0.05 average increase in accuracy (p < 0.001) when compared with the Log(T/C) ratio applied to the same array.

**Figure 3:**
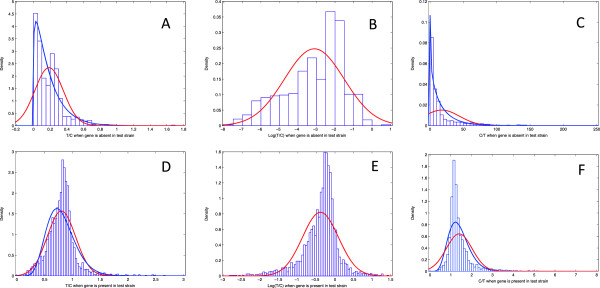
**Histograms of signal ratio values. A** and **D** represent histograms of the Test over Control ratio (T/C) values. **B** and **E** represent histograms of the logarithm of the Test over Control ratio (Log(T/C) values. **C** and **F** represent histograms of the Control over Test ratio (C/T) values. **A**, **B** and **C** histograms were drawn with ratio values of probes where the gene was absent in the test sample. **D**, **E** and **F** histograms were drawn with ratio values of probes where the gene was present in the test sample. Red lines are best fits of a Normal distribution to the drawn histogram. Blue lines are best fits of the Gamma distribution to the corresponding histograms. Due to the negative values of some Log (T/C) ratios, it was not possible to fit a Gamma distribution to the histograms in **B** and **E**. All the histograms where based on one *Streptococcus pneumoniae* microarray using the mix control.

To understand the impact of the ratio choice in the mixture model fit, we analyzed the distribution of ratio values in one of the pneumococcal arrays (Figure 3). It is possible to observe that if the gene is present in the test strain (T ≠ 0), all the three ratios can be represented in terms of location by Normal distributions and, in the case of T/C and C/T ratios, by Gamma distributions. However, the fitted distributions show more dispersion than the histograms, not achieving sufficiently high densities in the distribution peaks. The Normal distribution does not fit to any ratio distributions when the gene is not present in the test strain (T = 0). T/C and C/T ratios are better described by Gamma distributions when T = 0. This observation would predict better accuracies for the G mixture model with the T/C and C/T ratios. This prediction is correct for the T/C ratio, but the NU model leads to higher accuracies for the C/T and Log(C/T) ratios (Figure 1). The distributions of these two ratios when the T = 0 do not appear to be uniform. However, when compared with the corresponding distributions when T ≠ 0, they have much lower densities across a much larger range of values. This might explain the success of the NU model with C/T and Log (T/C) ratios. When T = 0, the distribution of T/C presents high densities, comparable with the densities of the T/C distribution when T ≠ 0. Additionally, for T = 0, the T/C values range from 0 to 1, presenting a large overlap with the T/C distribution when T ≠ 0. It is possibly this overlap that is responsible for the lower accuracies obtained when the T/C ratio is used.

**Figure 4:**
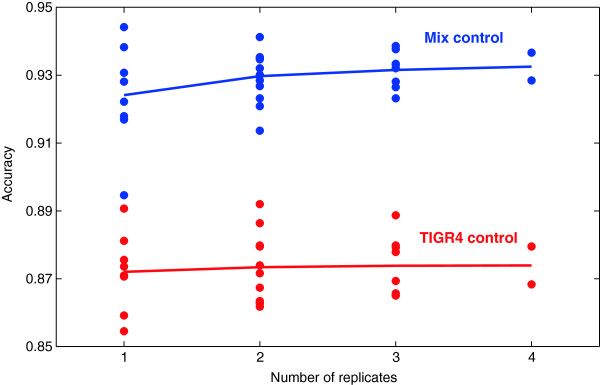
**Accuracy obtained with replicate averaging.** The blue (red) dots correspond to accuracies of different arrays or combinations of replicate arrays using the mix control (TIGR4 control). The blue (red) line represents the evolution of the mean accuracies with increasing replicate numbers for the mix (TIGR4) control.

Aiming to quantify the advantage in averaging replicate arrays, we combined the four available replicates of each hybridization into the possible sets of 2, 3 or 4 replicates and evaluated the accuracy of the resulting classifications, shown in Figure 4. In this evaluation we used the C/T ratios with the NU mixture model, as this option produced the best results without replication. Although there is a small increment in accuracy due to replication, no significant difference is found, justifying the use of just one or two replicates per sample in this particular application.

### Discussion

Other authors [2,3] have previously recognized that some methods applied to analyze aCGH experiments were inherited from microarray gene expression analysis, without specifically evaluating their adequacy for aCGH data. Our results highlight this problem, showing that loess normalization generally has no significant impact in gene classification as present or absent.

Logarithm transformation also has a negative impact when compared with the C/T ratio. In the analysis of gene expression, the aim is to identify genes that are either over or under expressed. The logarithm transformation is useful to make both types of changes quantitatively comparable. In aCGH experiments applied to bacterial genomes, genes are either present or absent (which includes genes with divergent sequences). The use of the C/T ratio expands the signal scale in the most interesting range for this type of analysis, facilitating the statistical segregation of signal distributions between present and absent genes. We should note that using the C/T ratio instead of log(T/C) (or of T/C ratio) only reverses the order of observed gene signals, without ranking changes. This implies that the optimal threshold separating present and absent genes with all three ratio variants will produce the same maximum accuracy. What changes is the capacity of the EM algorithm to adjust the mixture model to the available data, leading to better results with the C/T ratio. Previous studies have showed that fitting mixture models to aCGH data provided better result than several other methods [7,15]. Their comparison was based in the use of log(T/C) ratios. Our study reveals that the success of mixture model fitting in aCGH analysis can be improved with the use of the C/T ratio.

The present results also confirm our previous study [1], in which the advantages of the use of a mix control were first observed. We believe that the presence of a present signal in the control channel of all the spots in the array is important for the resulting classification obtained through the fitting of a mixture model to the observed C/T signal ratios. The choice of a mix or a single strain control is posed when working with arrays recognizing genes from multiple genomes. As the number of available genome in the array increases, the mix control may loose its superiority, as genes that are only present in one of the reference genomes will have an increasingly weak control signal, approaching 0. Other authors [2] have presented alternative methods for the analysis of bacterial aCGH experiments that use the signal distribution of the control channel independently of the test channel. These methods apparently reduce the need for a mix control. Nevertheless, our work shows that the mix control performs significantly better with an array designed for three reference genomes and analyzed with a mixture model approach.

Nowadays, comparative genomic studies in bacteria are migrating from the microarray technology to high throughput sequencing methods. This happens due to the decreases in the costs associated with sequencing, but also due to a gain in information: from present-absence status to a detection of diverging sequences. Still, some ongoing projects keep using microarrays, justifying the usefulness of the methodological comparison we are presenting [16-22]. Furthermore, our conclusions about the impact of the C/T ratio can justify the re-analysis of many already published and publicly available datasets, as we have demonstrated by analyzing the *S. aureus* arrays.

Our results must be interpreted taking into account some limitations of our study. We tested arrays from two species using a similar array design strategy and manufacturer. Additionally, only one normalization algorithm and two different mixture models were compared. Although we are confident that the observations are sufficiently general to apply in other contexts, we cannot anticipate the effect of different genomes, microarray platforms, normalization procedures or mixture models using different distributions. We could, for example, expand our study to include a higher number of different organisms, in order to generalize our conclusions. The advantages of the C/T ratio are not necessarily valid for every organism. Nevertheless, as our study shows that for two species the choice of ratio has a significant impact in classification accuracy, we can conclude that, when working with a different organism, it is worthy to conduct a similar study to optimize the signal ratio used for analysis.

Two aCGH analysis problems were not approached in our work. One is the detection of multiple gene copies. If there are more copies of a given gene in the genome of control strains than in the test strains, the gene can be erroneously classified as absent. From the array design it should be possible to identify probes targeting genes with multiple copies in the control strains, and perform a separate analysis in case they where classified as absent. Probes with a single copy in the control strains and multiple copies in the test strain will most likely be classified as present. A subsequent analysis of probes classified as present can be developed to quantify the number of copies, but it is expectable that as the copy number grows, there should be saturation in the fluorescence signal.

The second analysis problem is the quantification of sequence divergence. Our analysis returns a probability of gene presence in the test strain. When that probability has intermediate values (around 0.5), one can suspect of a divergent gene. However, the same values can be equally originated by excessive noise. Even if there are very low noise levels, a sequence divergence determined by microarray just implies a sequence difference along the region targeted by the microarray probe. The remaining sequence of the gene can be highly conserved or highly divergent. The method proposed by Snipen and colleagues [2] uses the signals from the control channel to adjust a quantitative function relating sequence divergence and signal intensity. Although this method gives a quantitative answer to this problem, it is still affected by the noise levels and by the diversity of divergent sequences in the control sample. Sequencing studies provide a better approach to detect and quantify divergent regions.

### Conclusions

We performed a comparison of several alternative methods to analyze bacterial aCGH datasets. All alternatives had in common the use of the EM algorithm to adjust a mixture model to the observed signal distributions. The results validated a previous study advocating the use of a strain mix as a control in aCGH experiments with multi-genome arrays, instead of a single strain control. Additionally, we found that loess normalization or the choice of mixture model distributions did not have a clear impact on the results. The method variant that induced a greater improvement in achieved classification accuracies was the use of a control-over-test signal ratio, which is the inverse of the ratio traditionally used to analyze this type of results.

### Availability of supporting data

The microarray datasets analyzed in this work are deposited in the Array Express public database with the following accession numbers: E-MEXP-1390, E-MEXP-2007.

## Competing interests

The authors declare that they have no competing interests.

## Author’ contributions

CES, FRP and LSC have performed the data analysis. MA, MR and FRP have conceived the study. All authors wrote, revised and approved the final manuscript.
